# Comparative genomics and phylogenomics of the genus *Glycyrrhiza* (Fabaceae) based on chloroplast genomes

**DOI:** 10.3389/fphar.2024.1371390

**Published:** 2024-03-07

**Authors:** Liwei Wu, Panhui Fan, Jiaying Cai, Chenxi Zang, Yulin Lin, Zhichao Xu, Zhengjun Wu, Wei Gao, Jingyuan Song, Hui Yao

**Affiliations:** ^1^ State Key Laboratory of Basis and New Drug Development of Natural and Nuclear Drugs, Institute of Medicinal Plant Development, Chinese Academy of Medical Sciences and Peking Union Medical College, Beijing, China; ^2^ College of Life Science, Northeast Forestry University, Harbin, China; ^3^ China Resources Sanjiu Medical & Pharmaceutical Co., Ltd., Shenzhen, China; ^4^ Engineering Research Center of Chinese Medicine Resources, Ministry of Education, Beijing, China

**Keywords:** *Glycyrrhiza*, Fabaceae, chloroplast genome, comparative genomics, phylogenomics

## Abstract

*Glycyrrhiza* (Fabaceae) species are rich in metabolites and widely used in medicine. Research on the chloroplast genome of *Glycyrrhiza* is important for understanding its phylogenetics, biogeography, genetic diversity, species identification, and medicinal properties. In this study, comparative genomics and phylogenomics of *Glycyrrhiza* were analyzed based on the chloroplast genome. The chloroplast genomes of six *Glycyrrhiza* species were obtained using various assembly and annotation tools. The final assembled chloroplast genome sizes for the six *Glycyrrhiza* species ranged from 126,380 bp to 129,115 bp, with a total of 109–110 genes annotated. Comparative genomics results showed that the chloroplast genomes of *Glycyrrhiza* showed typically lacking inverted repeat regions, and the genome length, structure, GC content, codon usage, and gene distribution were highly similar. Bioinformatics analysis revealed the presence of 69–96 simple sequence repeats and 61–138 long repeats in the chloroplast genomes. Combining the results of mVISTA and nucleotide diversity, four highly variable regions were screened for species identification and relationship studies. Selection pressure analysis indicated overall purifying selection in the chloroplast genomes of *Glycyrrhiza*, with a few positively selected genes potentially linked to environmental adaptation. Phylogenetic analyses involving all tribes of Fabaceae with published chloroplast genomes elucidated the evolutionary relationships, and divergence time estimation estimated the chronological order of species differentiations within the Fabaceae family. The results of phylogenetic analysis indicated that species from the six subfamilies formed distinct clusters, consistent with the classification scheme of the six subfamilies. In addition, the inverted repeat-lacking clade in the subfamily Papilionoideae clustered together, and it was the last to differentiate. Co-linear analysis confirmed the conserved nature of *Glycyrrhiza* chloroplast genomes, and instances of gene rearrangements and inversions were observed in the subfamily Papilionoideae.

## 1 Introduction

With the fervent progress of genome projects worldwide in the early 1990s, DNA sequences for phylogenetic studies accumulated rapidly. The integration of phylogenetics and genomics gave rise to phylogenomics and comparative genomics (Eisen, 1998). On the one hand, phylogenomics and comparative genomics utilize large-scale molecular data at the genomic level to determine the phylogenetic relationships between species. On the other hand, they integrate these phylogenetic relationships to investigate patterns and mechanisms of genome evolution (Eisen, 1998). The cell organelle genome in eukaryotes mainly includes all DNA molecules contained in mitochondria and plastids (including chloroplast, chromoplast, and leucoplast), serving as the primary carriers of cytoplasmic inheritance. With the emergence and development of DNA sequencing technologies, the organelle genome has become a crucial tool in research areas such as phylogenetics, biogeography, hybridization, and species identification in eukaryotes (Fujii et al., 2010; Smith, 2015). In plants, the chloroplast genome, characterized by a high copy number and moderate molecular substitution rate, has been utilized in systematic studies across various taxonomic ranks. It has achieved notable success in resolving phylogenetic relationships, particularly in addressing challenging taxonomic groups (Wolfe et al., 1987; Drouin et al., 2008).

The chloroplast genome is predominantly a closed circular DNA, typically ranging in size from 115 kb to 165 kb. In most angiosperms, it exhibits a relatively conserved quadripartite structure, consisting of two inverted repeat regions (IRs), a large single-copy region (LSC), and a small single-copy region (SSC), among which the two single-copy regions are separated by IRs (Jansen and Ruhlman, 2012). However, with the increasing number of reported chloroplast genomes, researchers have found varying degrees and types of size and structural variations in the chloroplast genome in some angiosperm families. The expansion, contraction, and even loss of the IRs are important factors influencing the size of the chloroplast genome (Jansen and Ruhlman, 2012). Research on the chloroplast genome of the Fabaceae family has mainly focused on the Papilionoideae subfamily. Compared with most angiosperms, the chloroplast genome structure in the Papilionoideae subfamily exhibits significant variation. One branch of this subfamily, encompassing all reported species, has experienced the loss of the IR regions in the chloroplast genome. Consequently, this branch is termed the inverted repeat-lacking clade (IRLC) (Wojciechowski et al., 2000). In the IRLC, chloroplast genomes have undergone extensive rearrangements, involving processes such as gene duplication, loss, and sequence inversion (Cai et al., 2008; Sveinsson and Cronk, 2014). For a long period, scholars believed that, except for the Papilionoideae subfamily, chloroplast genomes in other Fabaceae plants were highly conserved (Schwarz et al., 2015). However, subsequent research revealed a roughly 13 kb expansion of the IR into the SSC in the *Acacia* and *Inga* genera (Caesalpinioideae subfamily). Additionally, in the *Acacia* genus, IR underwent an approximately 2 kb contraction at the junction with the LSC (Dugas et al., 2015; Williams et al., 2015).

The genus *Glycyrrhiza*, belonging to the subfamily Papilionoideae of the family Fabaceae, is a perennial herbaceous plant. It is distributed worldwide, with main production areas in Asia and Europe, and a small number can be found in the tropical and subtropical regions of America and Africa. China is located in the central zone of *Glycyrrhiza* resources, and it is also the country with the largest consumption and export volume of *Glycyrrhiza* in the world (Zheng et al., 2015). According to the Flora of China, eight species of *Glycyrrhiza* exist in China, which can be classified into two sections based on the presence of glycyrrhizic acid: section *Glycyrrhiza* (*Glycyrrhiza uralensis* Fisch. ex DC., *Glycyrrhiza glabra* L., *Glycyrrhiza inflata* Batalin, *Glycyrrhiza aspera* Pall., and *Glycyrrhiza eglandulosa* X.Y.Li) and section *Pseudoglycyrrhiza* (*Glycyrrhiza pallidiflora* Maxim., *Glycyrrhiza squamulosa* Franch., and *Glycyrrhiza yunnanensis* S.H.Cheng & L.K.Tai ex P.C.Li) (Li et al., 2010). Among them, the species in section *Glycyrrhiza* contain glycyrrhizic acid, and they are often used as medicine. By contrast, the species in section *Pseudoglycyrrhiza* do not contain glycyrrhizic acid. *Glycyrrhiza* species are widely used in medicine, and the Chinese pharmacopoeia specifies that *G. uralensis*, *G. glabra*, and *G. inflata* are the primary source plants for medicinal materials, with their medicinal parts being the roots and rhizomes. It is known for their effects in tonifying the spleen and invigorating qi, clearing heat and detoxifying the body, relieving coughs, reducing phlegm, alleviating pain, and harmonizing the actions of various other medicinal herbs (Commission, 2020). The species in *Glycyrrhiza* are rich in metabolites. Among them, triterpenoid saponins and flavonoids are two important metabolites that determine the medicinal effects. Modern pharmacological research has discovered that species in *Glycyrrhiza* have various effects, including anti-inflammatory, analgesic, anticancer, antiviral, antioxidant, and hepatoprotective properties (Shin et al., 2007; Hawthorne and Gallagher, 2008; Shin et al., 2008; Ming and Yin, 2013). Besides their medicinal uses, *Glycyrrhiza* species are also applied as an additive and flavoring agent in industries such as food, tobacco, daily chemicals, and animal husbandry (Li et al., 2004). However, there has been ongoing controversy regarding the classification within the *Glycyrrhiza* genus, which has implications for the medicinal, industrial, and scientific applications of *Glycyrrhiza* species, as well as the collection and breeding of their germplasm resources. Non-medicinal species are sometimes sold in the market as medicinal species, and even some scientific studies on pharmacology or chemistry have used misidentified *Glycyrrhiza* materials (Duan et al., 2023).

To date, 23 species in *Glycyrrhiza* with chloroplast genomes have been published. However, comparative genomics studies of the chloroplast genome in *Glycyrrhiza* are scarce. Additionally, research on phylogenomics of the Fabaceae family using chloroplast genome data is limited, and no study has utilized chloroplast genomes to investigate the divergence times of Fabaceae species. This study performed the assembly and annotation of chloroplast genomes for six *Glycyrrhiza* species, namely, three from section *Glycyrrhiza* (*G. uralensis*, *G. glabra*, and *G. inflata*) and three from section *Pseudoglycyrrhiza* (*G. pallidiflora*, *G. squamulose*, and *G. yunnanensis*). The research explored the efficiency of different assembly and annotation tools on chloroplast genomes of *Glycyrrhiza* species and analyzed the codon usage, repeat sequences, gene selection pressure, and sequence variations of all species with published chloroplast genomes within the *Glycyrrhiza* genus. It aims to contribute to the understanding of the genetic background and phylogenetic evolution related to the phenotypes in the *Glycyrrhiza* genus. Additionally, an evolutionary tree was reconstructed for all tribes with published chloroplast genomes in the Fabaceae family, providing a systematic evolutionary analysis of Fabaceae species. The study also included analyses of differentiation times and sequence collinearity among Fabaceae species.

## 2 Materials and methods

### 2.1 Plant and DNA sources

Fresh leaves of *Glycyrrhiza uralensis* Fisch. ex DC., *Glycyrrhiza glabra* L., *Glycyrrhiza inflata* Batalin, and *Glycyrrhiza pallidiflora* Maxim. were obtained from the Beijing Medicinal Plant Garden, and *Glycyrrhiza squamulosa* Franch. and *Glycyrrhiza yunnanensis* S.H.Cheng & L.K.Tai ex P.C.Li were collected from Hengshui City in Hebei Province and Lijiang City in Yunnan Province, respectively. All samples were identified by Prof. Yulin Lin from the Institute of Medicinal Plant Development (IMPLAD). Voucher specimens were deposited in the herbarium at IMPLAD. Total DNA of the six species was extracted by a DNeasy Plant Mini Kit (Qiagen Co., Germany), and the DNA concentration and quality were assessed through Nanodrop 2000C spectrophotometry and electrophoresis in 1% (w/v) agarose gel, respectively.

### 2.2 DNA sequencing, assembly, and annotation

The DNA of six *Glycyrrhiza* species was used to generate libraries with average insert size of 500 bp and sequenced using Illumina Hiseq X in accordance with the standard protocol. Low-quality reads resulting from all samples were trimmed by Trimmomatic software (Bolger et al., 2014). GetOrganelle (Jin et al., 2020) and NOVOPlasty (Dierckxsens et al., 2017) were used to assemble the chloroplast genomes. GeSeq (Tillich et al., 2017), CPGAVAS2 (Shi et al., 2019), and PGA (Qu et al., 2019) software were used to annotate the sequences initially and correct them manually. The final complete chloroplast genomes of *G. uralensis*, *G. glabra*, *G. inflata*, *G. pallidiflora*, *G. squamulose*, and *G. yunnanensis* were submitted to GenBank and obtained the accession numbers of PP119344, PP119342, PP119340, PP119341, PP119343, and PP119345, respectively.

### 2.3 Structural analyses

Chloroplast genome maps were generated using Chloroplot (Zheng et al., 2020) and then manually corrected. CodonW software (Sharp and Li, 1987) was adopted to analyze the usage of codon. Simple sequence repeats (SSRs) were detected by MISA (Beier et al., 2017) with the definition of ≥10 repeat units for mononucleotide; ≥5 repeat units for dinucleotide; and ≥4 repeat units for trinucleotide; and ≥3 repeat units for tetranucleotide, pentanucleotide, and hexanucleotide SSRs. The long repeated sequences were detected by REPuter (Kurtz et al., 2001) with length of ≥30 bp and over 90% similarity between two copies.

### 2.4 Comparative and phylogenetic analyses

The chloroplast genomes of all published *Glycyrrhiza* species (Supplementary Table S1) were compared using the online genome comparison tool mVISTA software (Frazer et al., 2004). The nucleotide diversity values (Pi) of the chloroplast genomes were computed using DnaSP (Librado and Rozas, 2009). KaKs_Calculator v. 2.0 was used to determine the ratio of non-synonymous substitutions (Ka) and synonymous substitutions (Ks) (Wang et al., 2010). Chloroplast genome sequence homology and collinearity were analyzed using Mauve software (Darling et al., 2004). A total of 87 chloroplast genomes of Fabaceae species, including all tribes of Fabaceae with published chloroplast genomes (Supplementary Table S1), were used to construct a phylogenetic tree. MAFFT software (Katoh and Standley, 2013) was used to compare the common protein-coding genes shared by the chloroplast genomes of these Fabaceae species. Maximum likelihood (ML) analysis was carried out by IQ-TREE (Nguyen et al., 2015) based on these common protein-coding genes with a bootstrap of 1000 repetitions. ML analysis was conducted based on the GTR + F + R5 model. The MCMCTREE program in the PAML package (Yang, 2007) was used to estimate the differentiation time of Fabaceae. The topology of the legume ML tree was used to demarcate the differentiation time nodes, and the results were viewed and edited by FigTree software (http://tree.bio.ed.ac.uk/software/figtree/).

## 3 Results and discussion

### 3.1 Assembly and annotation of *Glycyrrhiza* chloroplast genomes

**FIGURE 1:**
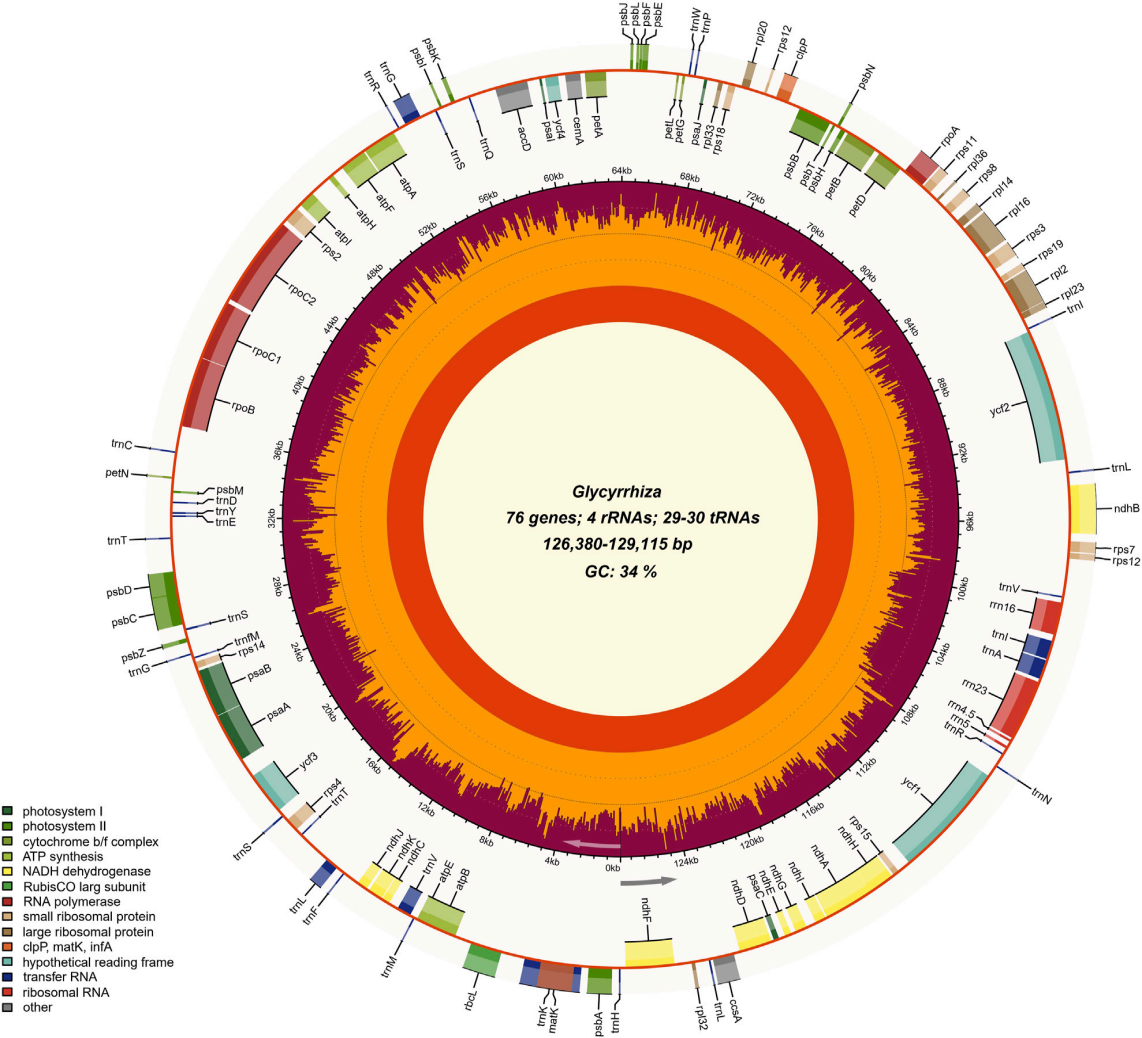
Chloroplast genome map of *Glycyrrhiza* species. The gradient GC content of the genome was plotted in the second circle with zero level based on the outer circle. The gene names were labeled on the outermost layer. The gene GC content was depicted with the proportion of shaded areas. Represented with arrows, the transcription directions for the inner and outer genes were listed clockwise and anticlockwise, respectively.

Correctly assembling complete chloroplast genomes ensures the reliability and reproducibility of research on the evolution of eukaryotic chloroplast genomes, as well as downstream studies based on chloroplast genomes. Currently, the most widely used tools for assembling organelle genomes are GetOrganelle and NOVOPlasty. This study employed GetOrganelle and NOVOPlasty for the assembly of six *Glycyrrhiza* species. The results revealed that the species successfully assembled using GetOrganelle were *G. uralensis*, *G. squamulosa*, and *G. yunnanensis*, whereas those successfully assembled using NOVOPlasty were *G. glabra*, *G. inflata*, *G. pallidiflora*, and *G. squamulosa*. The final assembled chloroplast genome sizes for the six *Glycyrrhiza* species ranged from 126,380 bp (*G. glabra*) to 129,115 bp (*G. yunnanensis*), with an overall GC content of approximately 34%. The coding sequences (CDS) accounted for the largest proportion, comprising around 52% of the total genome. The chloroplast genomes of the six *Glycyrrhiza* species showed a circular structure (Figure 1). Similar to the published chloroplast genomes of *Glycyrrhiza* species, these six chloroplast genomes also lacked the IRs. The success rates of chloroplast genome assembly for these six *Glycyrrhiza* species were similar between GetOrganelle and NOVOPlasty. However, a study based on testing with a publicly available dataset of reads from 50 plant species showed that, under slightly high computational resource consumption, the default parameter of GetOrganelle achieved a much higher complete circularity rate compared with the best parameter result of NOVOPlasty (Jin et al., 2020). In the benchmark study evaluating mainstream chloroplast genome assembly tools, GetOrganelle achieved significantly higher circularity and accuracy rates compared with other tools; thus, it was recommended as the optimal assembly tool (Freudenthal et al., 2020). Additionally, the output results from GetOrganelle included excellent visualization, allowing for an intuitive assessment of the assembly success without the need for additional verification. This feature significantly reduces the occurrence of false positives.

For chloroplast genome annotation, we employed the widely used organelle genome annotation tools, namely, GeSeq, CPGAVAS2, and PGA. The results indicated that the annotation effectiveness varied among different software tools for the six *Glycyrrhiza* species (Supplementary Table S2). GeSeq provides a comprehensive annotation of genes, and its real-time updated organelle genome database facilitates the selection of closely related species as references, which is crucial for the accuracy of chloroplast genome annotation. Combining these three annotation tools with manual correction, a total of 109–110 genes were annotated, including 76 protein-coding genes, 29–30 tRNAs, and 4 rRNAs, similar to the published *Glycyrrhiza* chloroplast genomes (Supplementary Table S3). Introns can accumulate more mutations than exons and play an important role in gene expression regulation (Kelchner, 2002). Previous studies have shown that introns can increase the expression level of foreign genes in eukaryotic genomes (Xu et al., 2003). In the six *Glycyrrhiza* species, there are a total of 16 genes with introns, with one gene (*ycf3*) having 2 introns, and the remaining 15 genes (*atpF*, *ndhA*, *ndhB*, *petB*, *petD*, *rpl16*, *rpl2*, *rpoC1*, *rps12*, *trnK-UUU*, *trnV-UAC*, *trnL-UAA*, *trnG-UCC*, *trnI-GAU*, and *trnA-UGC*) each having 1 intron (Supplementary Table S4). There were two special genes: *rps12* gene was a trans-splicing gene; *trnK-UUU* gene sequence contained the *matK* gene, similar to that of many other plants (Wu et al., 2021).

In summary, various chloroplast genome assembly and annotation tools yield varied results for different species or datasets, even within the same genus. Therefore, it is recommended to use multiple tools for chloroplast genome assembly and annotation, and the results from different tools should be combined to ensure the accuracy of chloroplast genome assembly and annotation.

### 3.2 Codon usage of *Glycyrrhiza* chloroplast genomes

Codon usage bias refers to the phenomenon that all synonymous codons in protein transcripts are not uniformly used to encode all amino acids in the protein except methionine and tryptophan (Jia and Xue, 2009). Given the difference in the degree of variation and selection pressure, the variation in compositional constraints between different genomes is a key factor in the formation of codon usage bias (Sharp and Li, 1987; Hershberg and Petrov, 2008; Paul et al., 2018). Codon usage bias can be used to investigate the evolutionary history of organisms, predict the expression level, and understand the evolutionary processes acting on genomes at the molecular level (Jia and Xue, 2009; Leffler et al., 2012).

**FIGURE 2:**
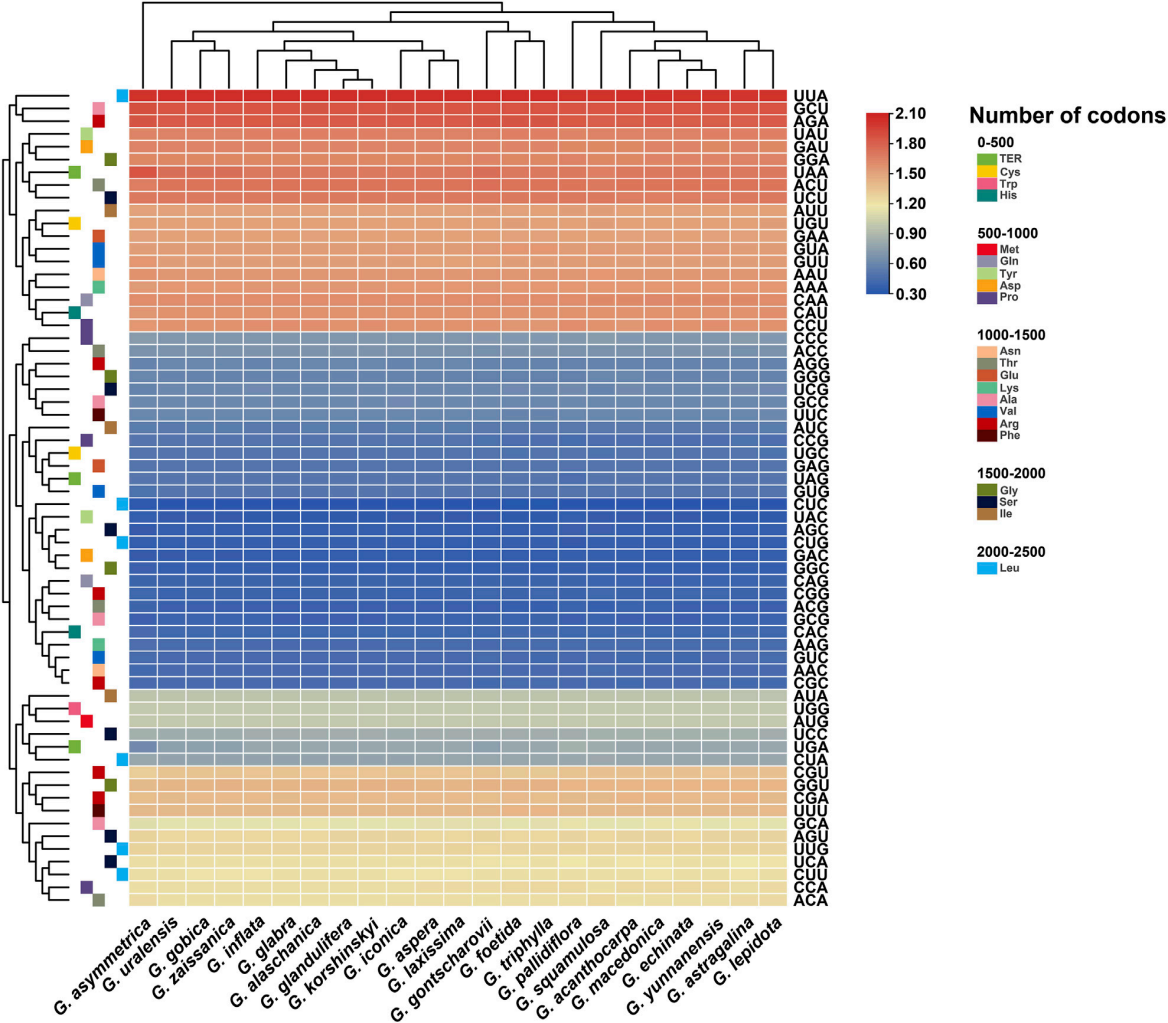
Heat map of RSCU values among *Glycyrrhiza* species.

The relative synonymous codon usage (RSCU) of the chloroplast genomes of all published *Glycyrrhiza* species was calculated based on all protein-coding genes (Figure 2). The results showed that the chloroplast genomes of *Glycyrrhiza* species contained 64 types of codons encoding 20 amino acids. Of all amino acid codons, leucine had the highest number of codons, whereas cysteine had the lowest number of codons. Thirty codons were found with an RSCU of >1, of which 29 were A/U-ending codons; 34 codons were found with an RSCU of ≤1, of which 31 were G/C-ending codons. Thus, compared with the G/C-ending codons, the chloroplast genome exhibited a greater bias toward the A/U-ending codons. The highest RSCU value was recorded for UUA and the lowest was recorded for CUC, which both encode leucine. In addition, the codon usage of the chloroplast genomes in section *Glycyrrhiza* was relatively close, whereas that of section *Pseudoglycyrrhiza* was close.

GC reflects the strength of directional mutation pressure, whereas GC3s is closely related to codon bias and used as an important basis for analyzing codon usage pattern (Shang et al., 2011; Zhao et al., 2016). GC and GC3s in the codons of these 23 chloroplast genomes were all less than 0.5, indicating that the chloroplast genomes of *Glycyrrhiza* species tended to use A/U bases and A/U-ending codons. Codon adaptation index values and effective number of codon values indicated a slight bias in codon usage in the *Glycyrrhiza* species. The frequency of optimal codons was relatively low. In addition, the hydrophobicity of the protein and the aromatic protein had little effect on codon usage bias (Table 1).

**TABLE 1:** Codon usage of the *Glycyrrhiza* species.

Species	GC3s	GC	CAI	ENc	Fop	Gravy	Aromo	L_sym	L_aa
*G. astragalina*	0.248	0.369	0.167	48.24	0.347	−0.047394	0.113196	21226	22121
*G. zaissanica*	0.247	0.368	0.167	48.16	0.347	−0.048145	0.113707	21239	22127
*G. foetida*	0.246	0.368	0.167	48.1	0.347	−0.048522	0.113764	21225	22116
*G. alaschanica*	0.247	0.368	0.167	48.17	0.347	−0.04951	0.11352	21249	22137
*G. korshinskyi*	0.247	0.368	0.167	48.17	0.347	−0.049031	0.113526	21239	22127
*G. squamulosa*	0.247	0.368	0.166	48.19	0.346	−0.046467	0.113602	21231	22121
*G. gontscharovii*	0.246	0.368	0.167	48.1	0.347	−0.048869	0.113367	21223	22114
*G. iconica*	0.247	0.368	0.167	48.16	0.347	−0.048692	0.113566	21249	22137
*G. acanthocarpa*	0.248	0.368	0.167	48.2	0.346	−0.049096	0.113727	21244	22132
*G. yunnanensis*	0.248	0.368	0.166	48.19	0.346	−0.047484	0.113626	21243	22134
*G. glandulifera*	0.247	0.368	0.167	48.17	0.347	−0.048778	0.113506	21243	22131
*G. asymmetrica*	0.248	0.368	0.166	48.25	0.346	−0.045656	0.113857	21285	22177
*G. uralensis*	0.247	0.368	0.167	48.17	0.347	−0.048963	0.113693	21232	22121
*G. echinata*	0.247	0.368	0.167	48.15	0.347	−0.046748	0.113728	21232	22123
*G. pallidiflora*	0.247	0.368	0.166	48.12	0.347	−0.051691	0.113367	21228	22114
*G. lepidota*	0.247	0.368	0.166	48.1	0.347	−0.047214	0.113471	21236	22129
*G. glabra*	0.247	0.368	0.167	48.17	0.347	−0.048563	0.113532	21238	22126
*G. triphylla*	0.247	0.368	0.167	48.12	0.348	−0.047907	0.113844	21228	22118
*G. aspera*	0.247	0.368	0.167	48.16	0.347	−0.049067	0.113561	21241	22129
*G. inflata*	0.247	0.368	0.167	48.14	0.347	−0.049559	0.113537	21237	22125
*G. macedonica*	0.247	0.368	0.167	48.11	0.346	−0.047017	0.113792	21237	22128
*G. laxissima*	0.247	0.368	0.167	48.17	0.348	−0.048728	0.113561	21241	22129
*G. gobica*	0.247	0.368	0.167	48.16	0.348	−0.048836	0.113627	21237	22125

GC3s, GC content at the synonymous third codon position; GC, total GC content; CAI, codon adaptation index; ENc, effective number of codons; Fop, frequency of optimal codons; Gravy, influence of protein hydrophobicity on codon usage bias; Aromo, influence of aromatic protein on codon usage bias; L_sym, number of synonymous codons; L_aa, total number of synonymous and non-synonymous codons.

### 3.3 SSRs and long repeat sequences

**FIGURE 3:**
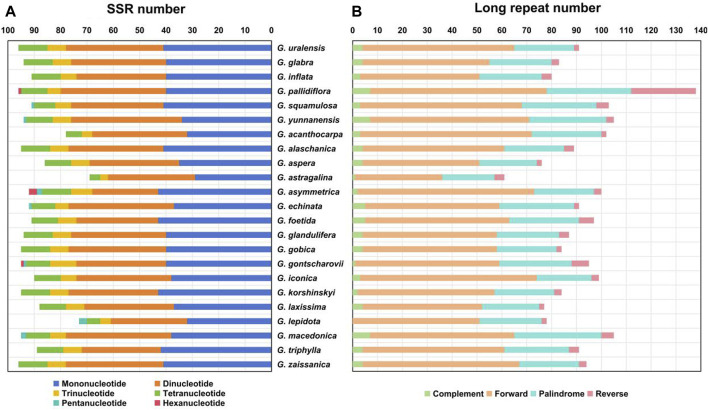
Repeat analysis of chloroplast genomes of *Glycyrrhiza* species. **(A)** SSR statistics of *Glycyrrhiza* species. **(B)** Long repeat statistics of *Glycyrrhiza* species. Different types of repeats are indicated by different colors.

SSRs are widely distributed in the chloroplast genome and usually used as important molecular markers for species identification (Yang et al., 2011; Jiao et al., 2012). A total of 69–96 SSRs were identified in the chloroplast genomes of the *Glycyrrhiza* species (Figure 3A). In addition, the base composition of the repeating motifs from mononucleotide repeats to tetranucleotide repeats, which existed in all *Glycyrrhiza* species, had a certain A-T base preference. In these SSRs, mononucleotide repeats were the largest in number, and they were found 31–43 times in these chloroplast genomes. A/T repeats were the most common mononucleotide repeats, whereas the majority of dinucleotide repeats, trinucleotide repeats, and tetranucleotide repeats comprised AT/AT, AAT/ATT, and AAAT/ATTT, respectively. These results were consistent with A-T enrichment in complete chloroplast genomes (Qian et al., 2013). Pentanucleotide repeats were found in *G. squamulosa*, *G. yunnanensis*, *Glycyrrhiza asymmetrica* Hub.-Mor., *Glycyrrhiza echinata* L., *Glycyrrhiza gontscharovii* Maslenn., *Glycyrrhiza lepidota* Pursh, and *Glycyrrhiza macedonica* Boiss. & Orph., and hexanucleotide repeats only existed in *G. pallidiflora*, *G. asymmetrica*, and *G. gontscharovii*.

Except for SSRs, some repetitive sequences with length ≥30 bp and sequence similarity ≥90% are called long repetitive sequences, including complement (C), forward (F), palindrome (P), and reverse (R). In *Glycyrrhiza* species, our results revealed 61 (*Glycyrrhiza astragalina* Gillies ex Hook. & Arn.)–138 (*G. pallidiflora*) long repeats, most of which were F (35–71) and P (21–35) repeats. No C repeat was found in the chloroplast genome of *G. lepidota*. The length of C and R repeats was mainly within the range of 30–39 bp (Figure 3B).

### 3.4 Variations in chloroplast genomes of *Glycyrrhiza* species

**FIGURE 4:**
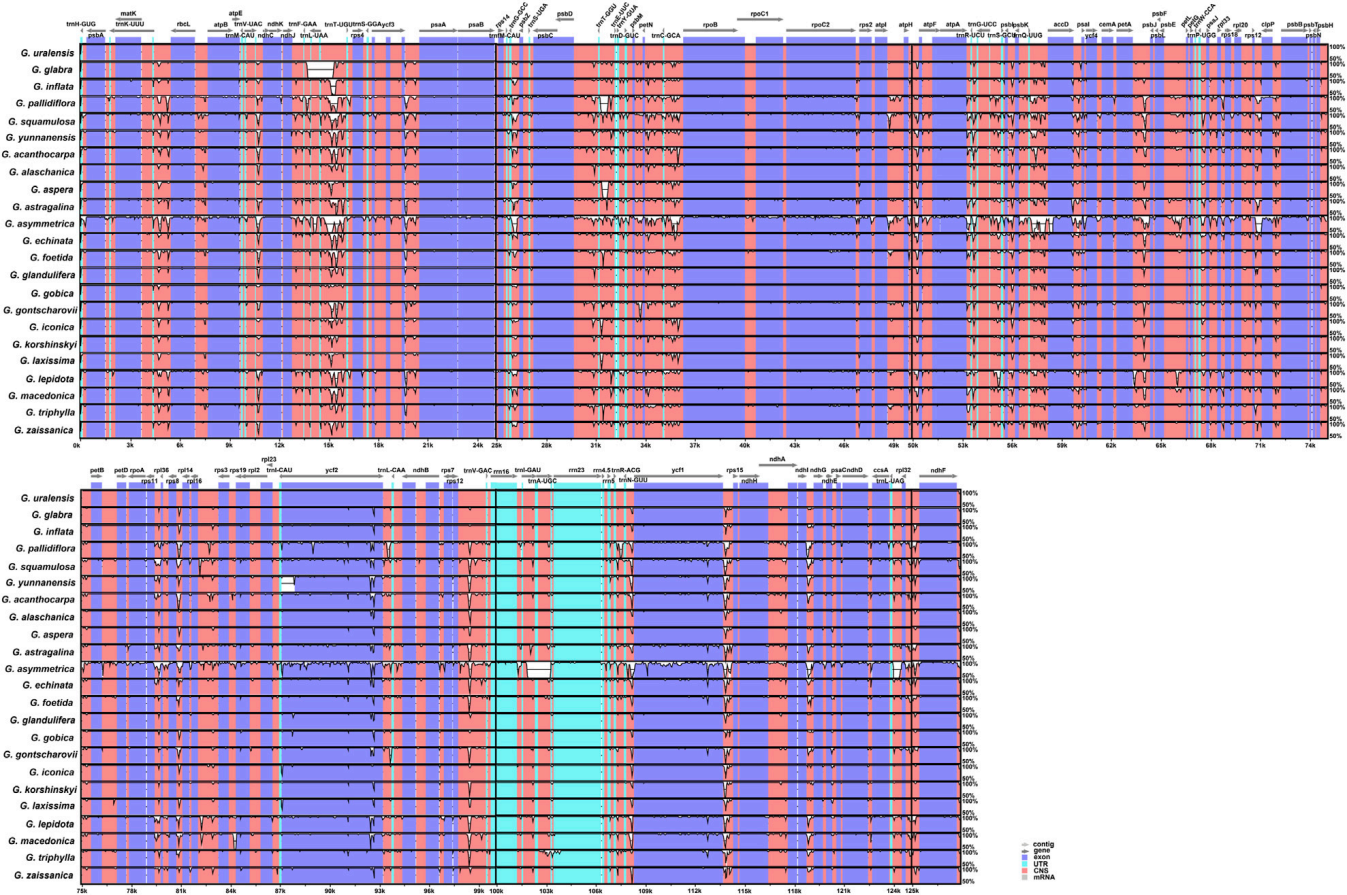
Global alignment of chloroplast genomes of 23 *Glycyrrhiza* species. The *x*-axis represents the coordinates in the chloroplast genome. The *y*-axis indicates the average percent identity of sequence similarity in the aligned regions, ranging between 50% and 100%.

In this study, the complete chloroplast genomes of all published *Glycyrrhiza* species were compared with the *G. uralensis* genome as the reference genome (Figure 4). Overall, the comparative genomic analysis revealed that the 23 *Glycyrrhiza* chloroplast genomes were relatively conserved. Intergenic spacers and intron regions showed more variations than protein-coding regions. Most protein-coding regions had a very high degree of conservation, and rRNA genes were highly conserved with almost no variation.

**FIGURE 5:**
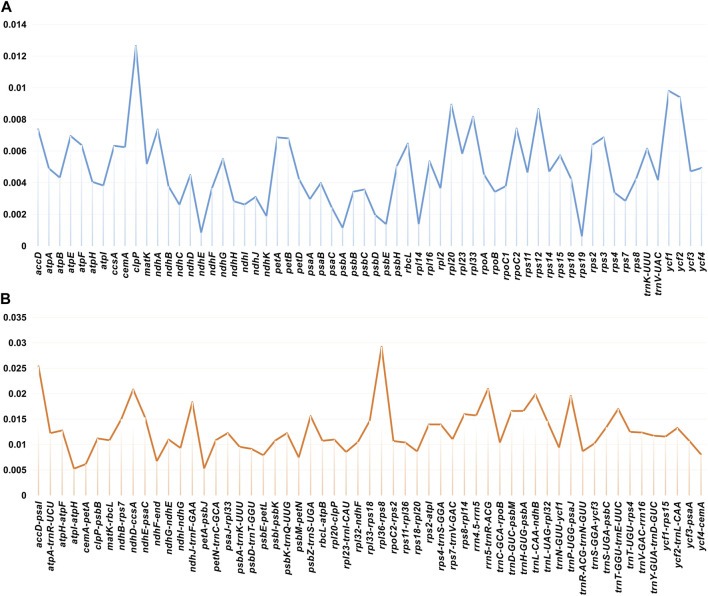
Nucleotide diversity of shared various regions with Pi > 0 in chloroplast genomes of 23 *Glycyrrhiza* species. **(A)** Pi values in the genes. **(B)** Pi values in the intergenic spacers.

Nucleotide diversity (Pi) of shared genes and intergenic spacers of the chloroplast genomes of the 23 *Glycyrrhiza* species were calculated. Figure 5 shows the intergenic spacers and genes with Pi > 0. The intergenic spacers had more polymorphisms (average Pi = 0.0127) than the gene regions (average Pi = 0.0049), consistent with mVISTA analysis. Combining mVISTA and Pi, four highly variable regions were screened out, namely, *accD*–*psaI*, *ndhD*–*ccsA*, *rpl36*–*rps8*, and *rrn5*–*trnR-ACG*, for species identification and relationship analysis.

### 3.5 Selective pressure analyses

Selective pressure refers to the external forces exerted on a species during the evolutionary process, driving the species to adapt to its natural environment. In genetics, ω = Ka/Ks represents the ratio between non-synonymous mutations (Ka) and synonymous mutations (Ks). In general, synonymous mutations are considered not subject to natural selection, whereas non-synonymous mutations are influenced by natural selection. ω > 1 indicates positive selection; ω = 1 implies neutral evolution, where no selection is acting; 0<ω < 1 is considered negative or purifying selection, with small ω values indicating high negative selection pressure (Wang et al., 2010).

**FIGURE 6:**
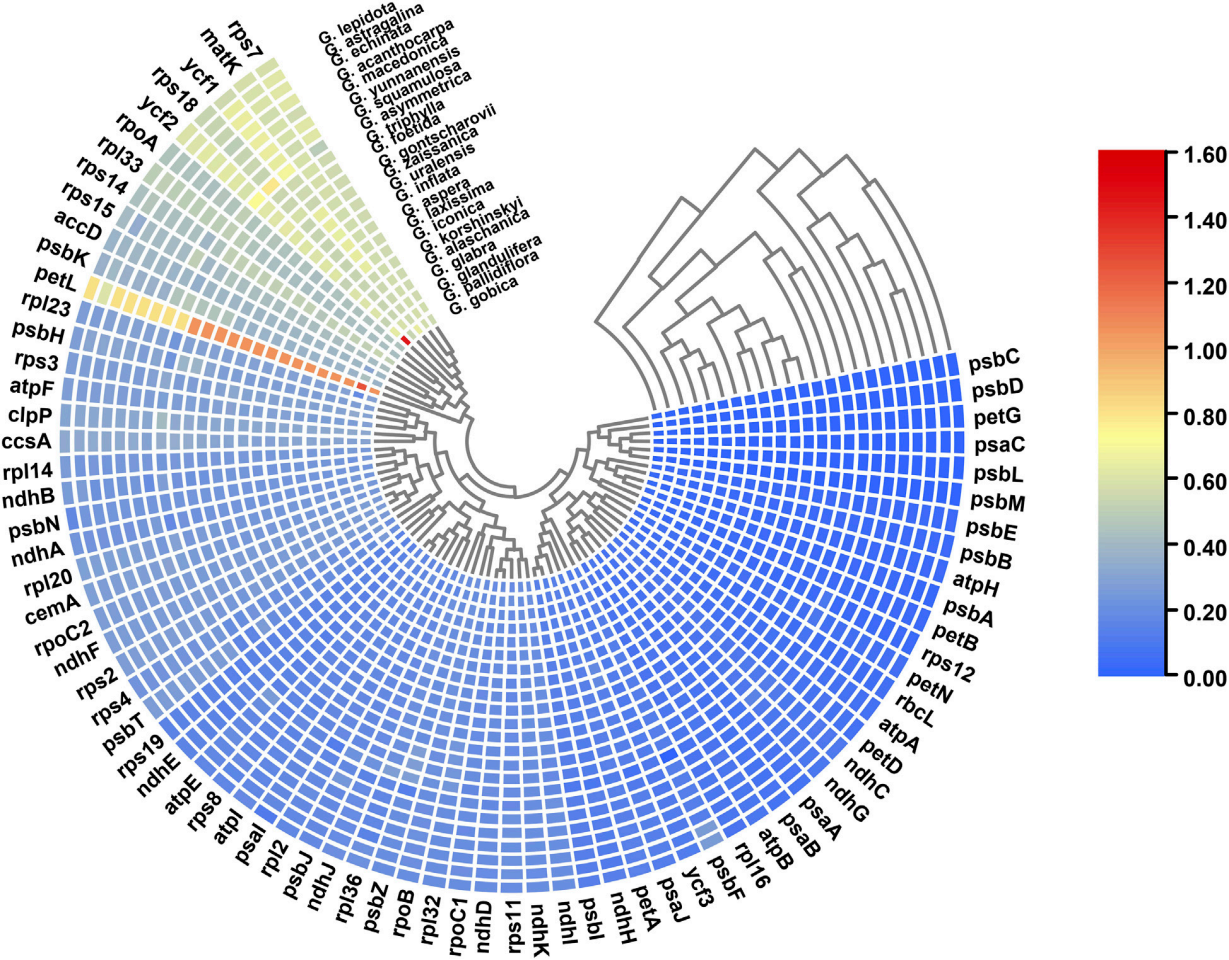
Selective pressure of genes in *Glycyrrhiza* chloroplast genomes.

The results of selective pressure analyses on *Glycyrrhiza* chloroplast genomes showed that almost all chloroplast genes in *Glycyrrhiza* species underwent negative or purifying selection, which indicated these genes were relatively conserved. Only *petL* in *G. glabra*, *G. pallidiflora*, *G. uralensis*, *G. inflata*, *Glycyrrhiza foetida* Desf., *Glycyrrhiza glandulifera* Waldst. & Kit., *Glycyrrhiza iconica* Hub.-Mor., *Glycyrrhiza laxissima* Vassilcz., *Glycyrrhiza triphylla* Fisch. & C.A.Mey., *G. aspera*, *Glycyrrhiza alaschanica* Grankina, *Glycyrrhiza gobica* Grankina, *G. gontscharovii*, *Glycyrrhiza zaissanica* Serg., and *Glycyrrhiza korshinskyi* Grig. and *ycf2* in *G. gobica* were positively selected genes, suggesting that some advantageous mutations were positively favored by selection (Figure 6). The positively selected genes may be associated with the adaptation of plants to low-light and low-temperature environments (Yang et al., 2023).

### 3.6 Phylogenetic analysis

Traditionally, the Fabaceae family is classified into three subfamilies based on the different floral morphologies: Mimosoideae, Caesalpinioideae, and Papilionoideae. Some traditional classification systems, such as the Cronquist system, elevate these three subfamilies to the rank of family (Taubert, 1894). However, molecular studies indicate that Mimosoideae and Papilionoideae are generally monophyletic, but Caesalpinioideae is a highly basal and paraphyletic group. Therefore, the practice of dividing Fabaceae into three subfamilies or three families does not adhere to the principle of monophyly (Taubert, 1894). Given the keen interest of the legume taxonomic community in the subordinal classification system, the Legume Phylogeny Working Group, composed of legume experts worldwide, published a consensual phylogenetic tree of the legume family in 2013, and three schemes for subfamily division were proposed (Group, 2013). Among these schemes, the 15-subfamily scheme retains the traditional concept of the Mimosoideae subfamily, whereas the Caesalpinioideae subfamily is split into several subfamilies (with over half of them distributed in China). The 6-subfamily scheme merges the Mimosoideae subfamily into a newly defined Caesalpinioideae, and it can avoid excessive splitting of the Caesalpinioideae subfamily. Considering that the species diversity of the traditional Mimosoideae and Caesalpinioideae in the Chinese region is not high, the 6-subfamily scheme is more convenient for Chinese scholars compared with the 15-subfamily scheme. In 2017, they officially adopted the 6-subfamily scheme, publishing the comprehensive Linnaean system for the classification of the legume family. They refined the hierarchical system below the subfamily level and above the genus level based on the research results accumulated in the academic community over the years (Group, 2017).

**FIGURE 7:**
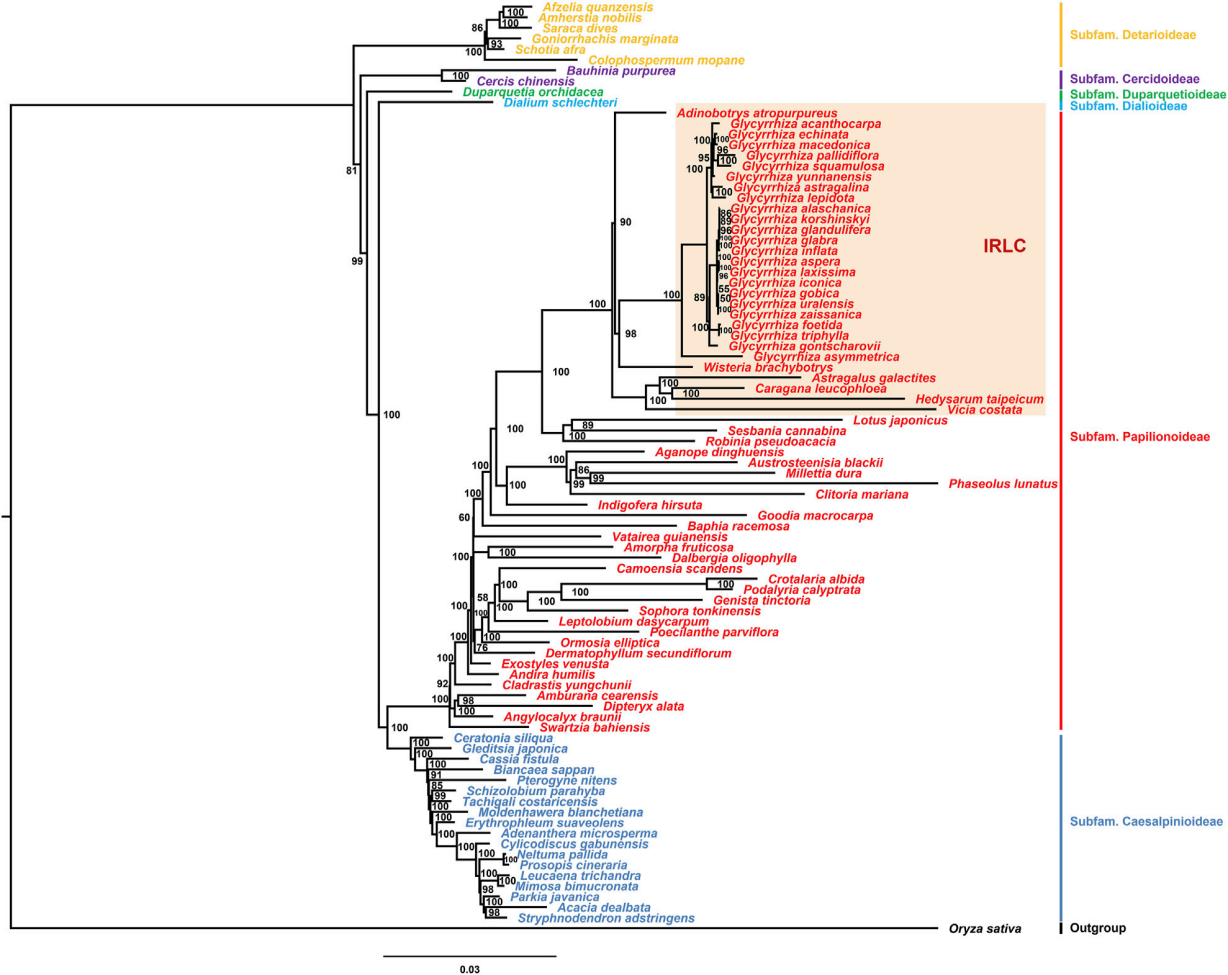
Phylogenetic tree constructed using the maximum likelihood (ML) method with a bootstrap of 1000 repetitions based on the chloroplast genomes of Fabaceae species. Numbers at nodes are values for bootstrap support.

The chloroplast genome is an important tool to explore the phylogeny of species (Hu et al., 2016). The Fabaceae family comprises 6 subfamilies and 79 tribes. Previous studies have focused on the phylogenetic relationship within the *Glycyrrhiza* genus (Jo et al., 2018; Jia et al., 2019; Duan et al., 2020; Jiang et al., 2020; Duan et al., 2023). Research on phylogenomics of the Fabaceae family using chloroplast genome data is limited. To date, chloroplast genomes of species from 65 tribes have been published. In this study, chloroplast genomes from species of these 65 tribes were used to construct a phylogenetic tree, including all published species of the *Glycyrrhiza* genus (Figure 7). The results indicated that species from the six subfamilies formed distinct clusters, consistent with the classification scheme of the six subfamilies. In the chloroplast genomes of Fabaceae plants, the absence of the IR regions was not unique to the *Glycyrrhiza* genus. Species lacking the IR regions were primarily distributed in various tribes, i.e., Glycyrrhizeae tribe, Adinobotryeae tribe, Wisterieae tribe, Hedysareae tribe, Caraganeae tribe, Astragaleae tribe, and Fabeae tribe in the Papilionoideae subfamily. The results of phylogenetic analysis showed that these tribes lacking the IR regions clustered together. Among them, the Glycyrrhizeae tribe clustered with the Wisterieae tribe and then with the Adinobotryeae tribe. The Hedysareae tribe, Caraganeae tribe, Astragaleae tribe, and Fabeae tribe formed a distinct cluster. In *Glycyrrhiza* species, the species of the section *Glycyrrhiza* and section *Pseudoglycyrrhiza*, distributed in China, respectively clustered together. The North American species *G. lepidota*, although had a lower content of glycyrrhizic acid (Hayashi et al., 2005) and was in another group, indicating that the groups containing glycyrrhizic acid were not monophyletic, consistent with previous studies (Duan et al., 2020). The Flora of China treats *G. alaschanica*, *G. korshinskyi*, and *G. gobica* as synonyms of *G. uralensis*; *G. glandulifera* as a synonym of *G. glabra*; and *G. laxissima* and *G. zaissanica* as synonyms of *G. aspera* (Li et al., 2010). However, according to the results of this study, only *G. gobica* and *G. uralensis* clustered together, *G. laxissima* and *G. aspera* clustered together, and others should be considered as independent species.

### 3.7 Divergence time estimation

**FIGURE 8:**
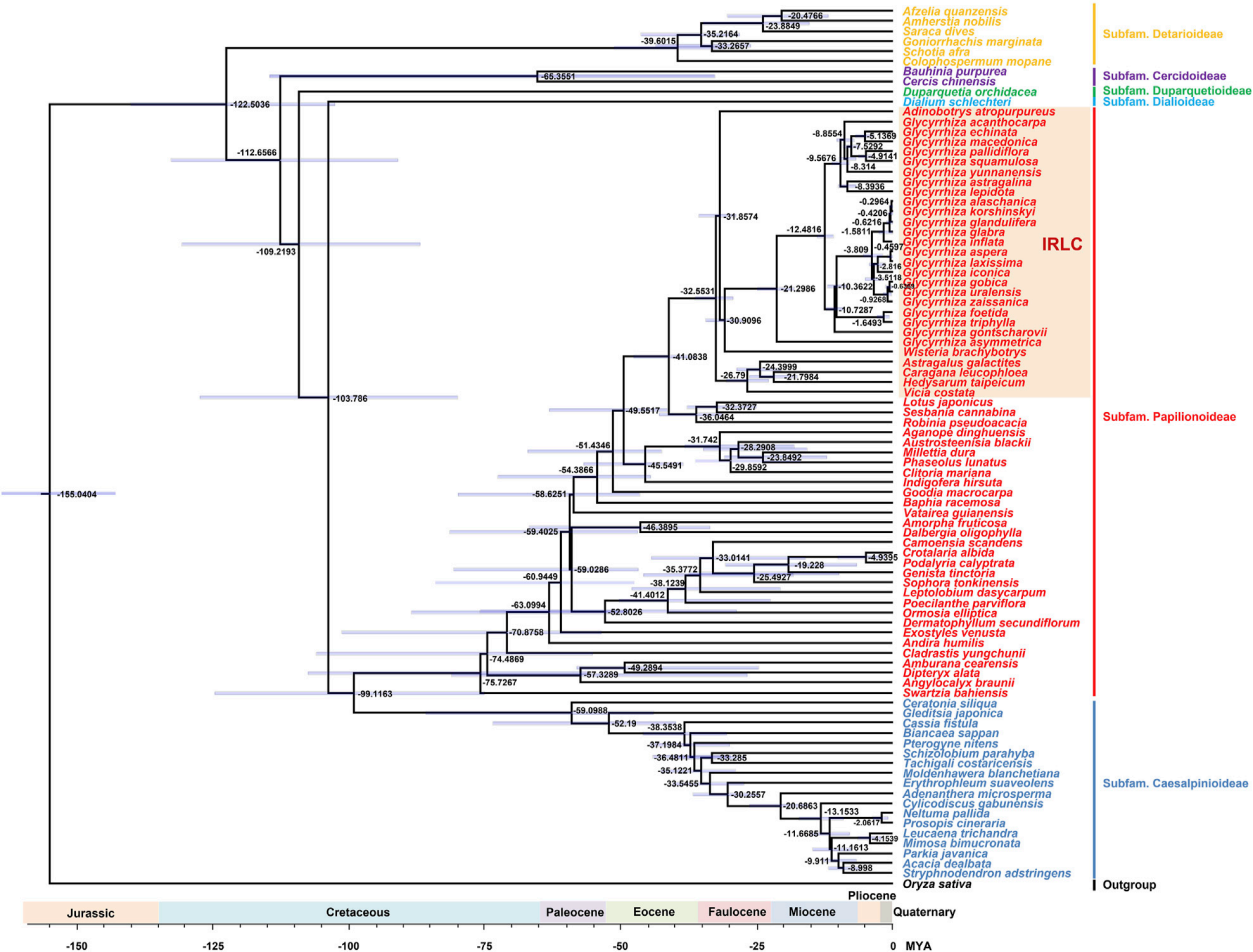
Divergence time estimation of Fabaceae species based on chloroplast genomes. The blue bars correspond to the 95% highest posterior density credibility intervals of age estimates. Numbers at nodes are values for divergence time (MYA).

In addition to being frequently used for phylogenetic analysis and species identification, the chloroplast genome is employed for studying species divergence times (Mo et al., 2022). This study explored the divergence times of Fabaceae species using the chloroplast genome (Figure 8). The results indicated that the subfamily Detarioideae was the first to diverge, followed by the subfamily Cercidoideae, subfamily Duparquetioideae, subfamily Dialioideae, subfamily Papilionoideae, and subfamily Caesalpinioideae. In the subfamily Papilionoideae, the IRLC population was the last to differentiate. In *Glycyrrhiza* species, the ones without glycyrrhizic acid differentiated earlier than those with glycyrrhizic acid. This result suggested that the common ancestor of *Glycyrrhiza* lacked glycyrrhizic acid, consistent with previous studies (Duan et al., 2023). Medicinal groups in the Eurasian continent share a common ancestor, and their descendants result from two rapid differentiation events within the last million years. This phenomenon has led to significant morphological over-diversification and taxonomic confusion within this group (Duan et al., 2023).

### 3.8 Co-linear analysis

**FIGURE 9:**
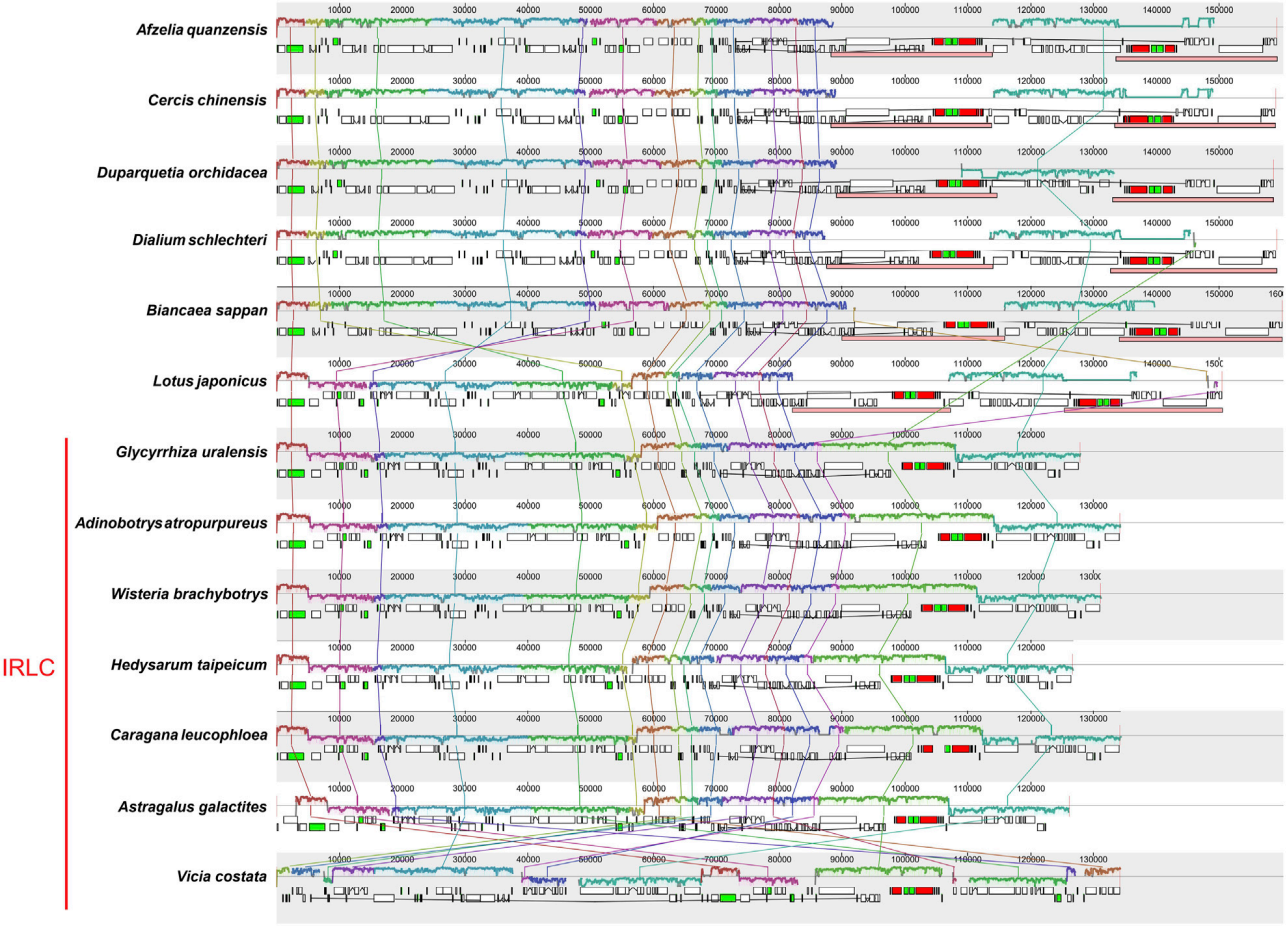
Co-linear analysis of Fabaceae chloroplast genomes. Local collinear blocks are represented by blocks of the same color connected by lines. The DNA fragments above the line correspond to the clockwise direction and those below the line are in the counterclockwise direction.

A co-linear analysis of the 23 *Glycyrrhiza* chloroplast genomes was conducted, and the *G. uralensis* genome was used as the reference genome. Results showed that the entire genome sequence was a homologous region with no big indels. The 23 chloroplast genomes connected with a line, indicating that the chloroplast genomes of *Glycyrrhiza* species were relatively conserved, and no rearrangement and inversion occurred in gene organization (Supplementary Figure S1). Additionally, a co-linear analysis was conducted on the chloroplast genomes of other species within the Fabaceae family (Figure 9). These species were from the six subfamilies: *Afzelia quanzensis* Welw. (subfamily Detarioideae), *Cercis chinensis* Bunge (subfamily Cercidoideae), *Duparquetia orchidacea* Baill. (subfamily Duparquetioideae), *Dialium schlechteri* Harms (subfamily Dialioideae), *Biancaea sappan* (L.) Tod. (subfamily Caesalpinioideae), and *Lotus japonicus* (Regel) K.Larsen (subfamily Papilionoideae), as well as all the tribes within the subfamily Papilionoideae that lack IR regions. Thus, the chloroplast genomes of *A. quanzensis*, *C. chinensis*, *D. orchidacea*, *D. schlechteri*, and *B. sappan* were relatively conservative, with minimal gene rearrangements and inversions. However, when compared with species in the subfamily Papilionoideae, especially *Vicia costata* Ledeb., the relative positions of some homologous blocks changed, which indicated rearrangements in the sequence. Additionally, homologous blocks below the line indicated reverse complementary alignments, also known as inversion blocks, suggesting possible inversion events. Species with gene rearrangements and inversions may be prone to the structural mutations in DNA strands and accumulate constantly due to the low GC content in their chloroplast genomes under the long evolutionary history (Ding et al., 2021).

## 4 Conclusion

This study focused on the comparative genomics and phylogenomics of *Glycyrrhiza* based on the chloroplast genome. Comparative genomics results showed that the chloroplast genomes of *Glycyrrhiza* had typically lacking IR regions, and the genome length, structure, GC content, codon usage, and gene distribution were highly similar. Selection pressure analysis indicated overall purifying selection in the chloroplast genomes of *Glycyrrhiza*, and some positively selected genes were potentially linked to environmental adaptation. The results of phylogenetic analysis and divergence time estimation showed that species from the six subfamilies formed distinct clusters, consistent with the classification scheme of the six subfamilies. The subfamily Detarioideae was the first to diverge, followed by the subfamily Cercidoideae, subfamily Duparquetioideae, subfamily Dialioideae, subfamily Papilionoideae, and subfamily Caesalpinioideae. In addition, the IRLC population in the subfamily Papilionoideae clustered together, and it was the last to differentiate. The groups containing glycyrrhizic acid in *Glycyrrhiza* were not monophyletic, and the common ancestor of *Glycyrrhiza* lacked glycyrrhizic acid. Co-linear analysis confirmed the conserved nature of *Glycyrrhiza* chloroplast genomes, as well as instances of gene rearrangements and inversions in the subfamily Papilionoideae.

## Data Availability

The datasets presented in this study can be found in online repositories. The names of the repository/repositories and accession number(s) can be found in the article/Supplementary Material. The data presented in the study are deposited in the GenBank repository (www.ncbi.nlm.nih.gov/genbank), accession number PP119344, PP119342, PP119340, PP119341, PP119343, and PP119345.
