# Supplementary material for: Comparative genomics and phylogenomics of the genus Glycyrrhiza (Fabaceae) based on chloroplast genomes
Source: Front Pharmacol. 2024 Mar 7;15:1371390. doi: 10.3389/fphar.2024.1371390 (PMC10955637; doi:10.3389/fphar.2024.1371390)
Supplement: Supplementary file 1 [file DataSheet1.PDF]

Supplementary Material

1. Supplementary Figures and Tables

1.1 Supplementary Figures

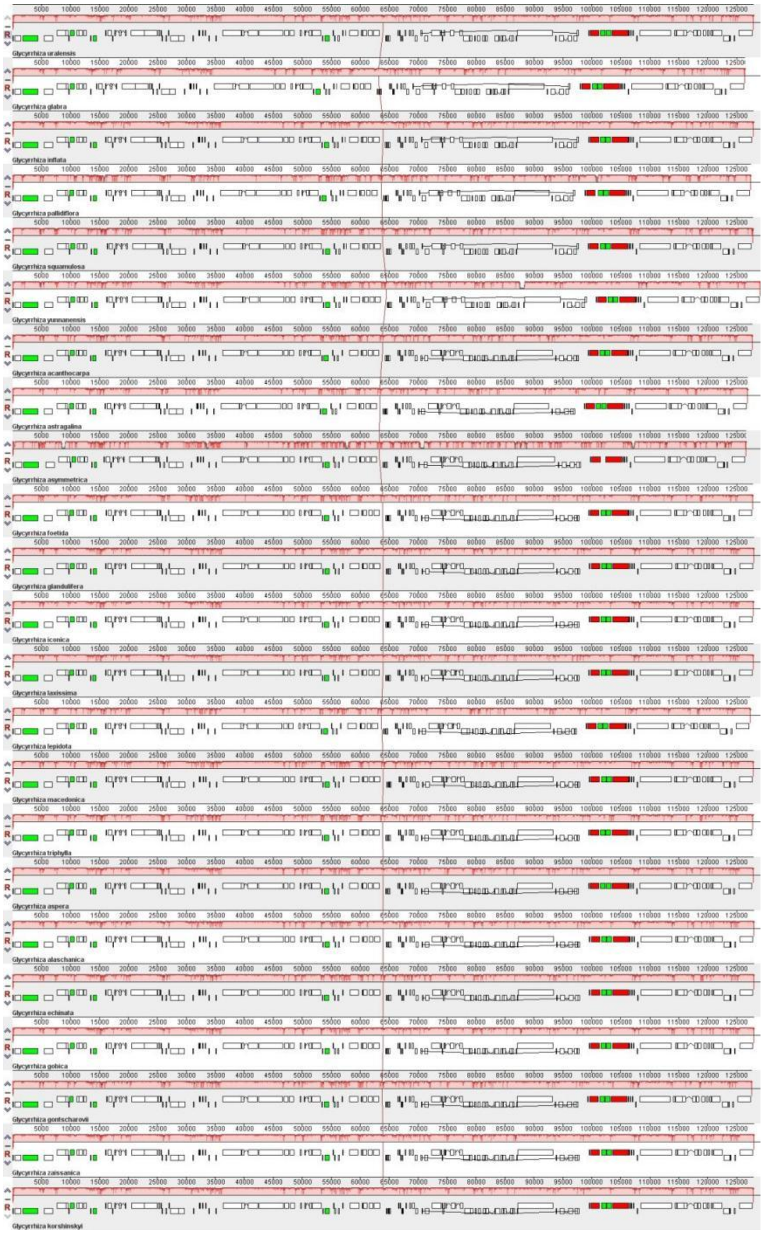

Supplementary Figure S1. Co-linear analysis of *Glycyrrhiza* chloroplast genomes. Local collinear blocks are represented by blocks of the same color connected by lines. The DNA fragments above the line correspond to the clockwise direction and those below the line are in the counterclockwise direction.

1.2 Supplementary Tables

Supplementary Table S1. Information of chloroplast genomes used in Fabaceae

Species	NCBI accession number	Length (bp)	GC content (%)
Glycyrrhiza uralensis	PP119344	127862	34.3
Glycyrrhiza glabra	PP119342	126380	34.3
Glycyrrhiza inflata	PP119340	127840	34.3
Glycyrrhiza pallidiflora	PP119341	127458	34.1
Glycyrrhiza squamulosa	PP119343	127738	34.2
Glycyrrhiza yunnanensis	PP119345	129115	34.3
Glycyrrhiza alaschanica	NC065140	127990	34.3
Glycyrrhiza korshinskyi	NC065145	127894	34.3
Glycyrrhiza gobica	NC065142	127970	34.3
Glycyrrhiza glandulifera	MT120773	127982	34.3
Glycyrrhiza laxissima	MT120777	127885	34.3
Glycyrrhiza zaissanica	NC065144	127984	34.3
Glycyrrhiza aspera	NC048509	127831	34.3
Glycyrrhiza echinata	NC065141	127993	34.2
Glycyrrhiza iconica	MT120774	127906	34.3
Glycyrrhiza foetida	MT120764	127817	34.3
Glycyrrhiza triphylla	MT120803	127767	34.2
Glycyrrhiza gontscharovii	NC065143	127616	34.3
Glycyrrhiza macedonica	MT120782	127894	34.3
Glycyrrhiza acanthocarpa	MT120750	127694	34.3
Glycyrrhiza astragalina	MT120755	126903	34.5
Glycyrrhiza lepidota	MT120778	127331	34.4
Glycyrrhiza asymmetrica	MT120759	126611	34.2
Cercis chinensis	MZ128523	158999	36.2
Bauhinia purpurea	NC061218	156099	36.4
Schotia afra	NC047328	158997	36.4
Goniorrhachis marginata	NC047394	158362	36.5
Colophospermum mopane	NC047388	157971	35.9
Saraca dives	NC071827	159627	36.2
Afzelia quanzensis	NC047329	159275	36
Amherstia nobilis	NC047327	158553	36.5
Duparquetia orchidacea	NC047346	158773	36.7
Dialium schlechteri	NC047326	159190	36.2
Ceratonia siliqua	NC026678	156367	36.7
Gleditsia japonica	NC070139	170796	33.9
Pterogyne nitens	NC047350	157422	36.7
Cassia fistula	NC065246	161724	36.1
Biancaea sappan	NC049085	160192	36
Tachigali costaricensis	NC047339	169313	36.8
Schizolobium parahyba	NC047316	159973	36.3
Moldenhawera blanchetiana	NC047348	160196	36.5
Erythrophleum suaveolens	NC047374	160344	36.5
Adenanthera microsperma	NC034986	159389	36.4

Cylicodiscus gabunensis	MZ274089	161494	36.2
Prosopis cineraria	NC049133	163677	35.9
Neltuma pallida	OR178743	162381	36
Leucaena trichandra	NC028733	164692	35.6
Parkia javanica	NC034989	161681	35.9
Stryphnodendron adstringens	NC044627	162169	35.9
Mimosa bimucronata	NC061740	167340	35
Acacia dealbata	NC034985	174217	35.4
Angylocalyx braunii	NC047392	162989	35.6
Dipteryx alata	NC067513	162568	34.2
Amburana cearensis	NC067514	161700	35.4
Swartzia bahiensis	MW628941	160100	35.7
Cladrastis yungchunii	NC058316	163205	36.9
Exostyles venusta	MW628960	155942	36.1
Vatairea guianensis	NC047363	155637	35.8
Andira humilis	NC067518	158920	36.1
Dermatophyllum secundiflorum	NC047349	159674	36.4
Ormosia elliptica	NC081487	169797	36.1
Poecilanthe parviflora	NC047393	160576	35.5
Leptolobium dasycarpum	MW628950	162998	35.8
Camoensia scandens	ON009081	127811	35.5
Sophora tonkinensis	NC042688	155640	36.4
Podalyria calyptrata	NC057458	152992	36.6
Crotalaria albida	NC061361	152743	36.6
Genista tinctoria	NC047375	152222	36.8
Amorpha fruticosa	NC047310	158245	36
Dalbergia oligophylla	NC072935	159310	36.4
Baphia racemosa	NC047397	156237	35.7
Goodia macrocarpa	NC047331	154333	35.9
Indigofera hirsuta	NC079834	159873	35.6
Aganope dinghuensis	NC054355	143690	35.3
Clitoria mariana	NC067531	152364	34
Austrosteenisia blackii	NC057276	152551	35
Millettia dura	NC057456	152714	34.9
Phaseolus lunatus	NC060403	150902	35.5
Sesbania cannabina	NC057145	153978	35.6
Lotus japonicus	NC002694	150519	36
Robinia pseudoacacia	NC026684	154835	35.8
Adinobotrys atropurpureus	NC068230	134267	34
Wisteria brachybotrys	NC060810	131179	34.3
Hedysarum taipeicum	MK426698	126699	35.1
Caragana leucophloea	NC077533	134240	34.7
Astragalus galactites	NC058825	126117	34
Vicia costata	NC057995	134184	34.9

Supplementary Table S2. Numbers of genes annotated by different tools

Species	Gene type	CPGAVAS2	PGA	Geseq
G. uralensis	protein-coding gene	75	76	76
	tRNA	29	30	30
	rRNA	4	4	4
	total	108	110	110
G. glabra	protein-coding gene	75	76	76
	tRNA	28	29	29
	rRNA	4	0	4
	total	107	105	109
G. inflata	protein-coding gene	74	75	76
	tRNA	29	30	30
	rRNA	4	4	4
	total	107	109	110
G. pallidiflora	protein-coding gene	75	76	76
	tRNA	28	29	30
	rRNA	4	0	4
	total	107	105	110
G. squamulosa	protein-coding gene	75	76	76
	tRNA	29	30	30
	rRNA	4	0	4
	total	108	106	110
G. yunnanensis	protein-coding gene	75	76	76
	tRNA	29	30	30
	rRNA	4	0	4
	total	108	106	110

Supplementary Table S3. Basic characteristics of the chloroplast genomes of *Glycyrrhiza*

Species	NCBI accession number	Length (bp)	GC content (%)	Number of genes		
				protein-coding genes	tRNAs	rRNAs
Glycyrrhiza uralensis	PP119344	127862	34.3	76	30	4
Glycyrrhiza glabra	PP119342	126380	34.3	76	29	4
Glycyrrhiza inflata	PP119340	127840	34.3	76	30	4
Glycyrrhiza pallidiflora	PP119341	127458	34.1	76	30	4
Glycyrrhiza squamulosa	PP119343	127738	34.2	76	30	4
Glycyrrhiza yunnanensis	PP119345	129115	34.3	76	30	4
Glycyrrhiza alaschanica	NC065140	127990	34.3	76	30	4
Glycyrrhiza korshinskyi	NC065145	127894	34.3	76	30	4
Glycyrrhiza gobica	NC065142	127970	34.3	76	30	4
Glycyrrhiza glandulifera	MT120773	127982	34.3	76	30	4
Glycyrrhiza laxissima	MT120777	127885	34.3	76	30	4
Glycyrrhiza zaissanica	NC065144	127984	34.3	76	30	4
Glycyrrhiza aspera	NC048509	127831	34.3	76	30	4
Glycyrrhiza echinata	NC065141	127993	34.2	76	30	4
Glycyrrhiza iconica	MT120774	127906	34.3	76	30	4
Glycyrrhiza foetida	MT120764	127817	34.3	76	30	4
Glycyrrhiza triphylla	MT120803	127767	34.2	76	30	4
Glycyrrhiza gontscharovii	NC065143	127616	34.3	76	30	4
Glycyrrhiza macedonica	MT120782	127894	34.3	76	30	4
Glycyrrhiza acanthocarpa	MT120750	127694	34.3	76	30	4
Glycyrrhiza astragalina	MT120755	126903	34.5	76	30	4
Glycyrrhiza lepidota	MT120778	127331	34.4	76	30	4
Glycyrrhiza asymmetrica	MT120759	126611	34.2	76	28	4

Supplementary Table S4-1. Information of genes with introns in the chloroplast genome of *Glycyrrhiza uralensis*

Genes	Direction	Start and stop sites of exons		
		Exon I	Exon II	Exon III
rps12	(-)	70509 – 70622	97417 – 97686	
rpoC1	(+)	39536 – 39965	40660 – 42284	
atpF	(+)	50380 – 50523	51189 – 51599	
petB	(+)	74737 – 74742	75551 – 76192	
petD	(+)	76434 – 76441	77149 – 77623	
rpl16	(-)	83077 – 83085	81586 – 81984	
rpl2	(-)	85787 – 86183	84643 – 85076	
ndhB	(-)	95813 – 96535	94372 – 95127	
ndhA	(+)	115833 – 116383	117594 – 118134	
trnK-UUU	(-)	4331 – 4367	1765 – 1799	
trnV-UAC	(+)	9924 – 9962	10535 – 10571	
trnL-UAA	(-)	14429 – 14465	13883 – 13932	
trnG-UCC	(-)	54611 – 54633	53876 – 53926	
trnI-GAU	(+)	101504 – 101545	102324 – 102358	
trnA-UGC	(+)	102427 – 102464	103275 – 103311	
ycf3	(+)	17613 – 17736	18450 – 18677	19412 – 19566

Supplementary Table S4-2. Information of genes with introns in the chloroplast genome of *Glycyrrhiza glabra*

Genes	Direction	Start and stop sites of exons		
		Exon I	Exon II	Exon III
rps12	(-)	69077 – 69190	95962 – 96231	
rpoC1	(+)	37993 – 38422	39117 – 40741	
atpF	(+)	48820 – 48986	49627 – 50038	
petB	(+)	73301 – 73306	74116 – 74757	
petD	(+)	74999 – 75006	75714 – 76188	
rpl16	(-)	81648 – 81656	80157 – 80555	
rpl2	(-)	84358 – 84754	83214 – 83647	
ndhB	(-)	94358 – 95080	92917 – 93672	
ndhA	(+)	114353 – 114903	116114 – 116654	
trnK-UUU	(-)	4331 – 4367	1765 – 1799	
trnV-UAC	(+)	9932 – 9970	10543 – 10579	
trnG-UCC	(-)	53053 – 53075	52318 – 52368	
trnI-GAU	(+)	100048 – 100089	100868 – 100902	
trnA-UGC	(+)	100971 – 101008	101819 – 101855	
ycf3	(+)	16024 – 16147	16863 – 17090	17825 – 17979

Supplementary Table S4-3. Information of genes with introns in the chloroplast genome of *Glycyrrhiza inflata*

Genes	Direction	Start and stop sites of exons		
		Exon I	Exon II	Exon III
rps12	(-)	70516 – 70629	97402 – 97671	
rpoC1	(+)	39459 – 39888	40583 – 42207	
atpF	(+)	50298 – 50464	51105 – 51516	
petB	(+)	74740 – 74745	75555 – 76196	
petD	(+)	76438 – 76445	77153 – 77627	
rpl16	(-)	83088 – 83096	81597 – 81995	
rpl2	(-)	85798 – 86194	84654 – 85087	
ndhB	(-)	95798 – 96520	94357 – 95112	
ndhA	(+)	115808 – 116358	117569 – 118109	
trnK-UUU	(-)	4325 – 4361	1765 – 1799	
trnV-UAC	(+)	9927 – 9965	10538 – 10574	
trnL-UAA	(-)	14431 – 14467	13885 – 13934	
trnG-UCC	(-)	54525 – 54547	53790 – 53840	
trnI-GAU	(+)	101488 – 101529	102308 – 102342	
trnA-UGC	(+)	102411 – 102448	103259 – 103295	
ycf3	(+)	17584 – 17707	18422 – 18649	19390 – 19544

Supplementary Table S4-4. Information of genes with introns in the chloroplast genome of *Glycyrrhiza pallidiflora*

Genes	Direction	Start and stop sites of exons		
		Exon I	Exon II	Exon III
rps12	(-)	70160 – 70273	96757 – 97026	
rpoC1	(+)	39104 – 39533	40222 – 41846	
atpF	(+)	49927 – 50093	50735 – 51146	
petB	(+)	74331 – 74336	75137 – 75778	
petD	(+)	76020 – 76027	76734 – 77208	
rpl16	(-)	82522 – 82530	81067 – 81465	
rpl2	(-)	85224 – 85622	84087 – 84521	
ndhB	(-)	95135 – 95857	93694 – 94449	
ndhA	(+)	115355 – 115905	117115 – 117655	
trnK-UUU	(-)	4364 – 4400	1802 – 1836	
trnV-UAC	(+)	9890 – 9927	10501 – 10537	
trnL-UAA	(-)	14431 – 14467	13885 – 13934	
trnG-UCC	(-)	54125 – 54147	53391 – 53441	
trnI-GAU	(+)	101098 – 101139	101911 – 101945	
trnA-UGC	(+)	102014 – 102051	102847 – 102883	
ycf3	(+)	17665 – 17788	18505 – 18732	19470 – 19624

Supplementary Table S4-5. Information of genes with introns in the chloroplast genome of *Glycyrrhiza squamulosa*

Genes	Direction	Start and stop sites of exons		
		Exon I	Exon II	Exon III
rps12	(-)	70661 – 70774	97404 – 97673	
rpoC1	(+)	39718 – 40147	40843 – 42467	
atpF	(+)	50453 – 50619	51265 – 51676	
petB	(+)	74864 – 74869	75669 – 76310	
petD	(+)	76548 – 76555	77263 – 77737	
rpl16	(-)	83096 – 83104	81666 – 82064	
rpl2	(-)	85802 – 86200	84666 – 85100	
ndhB	(-)	95798 – 96520	94365 – 95120	
ndhA	(+)	115689 – 116239	117439 – 117979	
trnK-UUU	(-)	4319 – 4355	1753 – 1787	
trnV-UAC	(+)	10013 – 10050	10624 – 10660	
trnL-UAA	(-)	14539 – 14575	13993 – 14042	
trnG-UCC	(-)	54723 – 54745	53972 – 54022	
trnI-GAU	(+)	101436 – 101477	102257 – 102291	
trnA-UGC	(+)	102360 – 102397	103201 – 103237	
ycf3	(+)	17818 – 17941	18659 – 18886	19626 – 19780

Supplementary Table S4-6. Information of genes with introns in the chloroplast genome of *Glycyrrhiza yunnanensis*

Genes	Direction	Start and stop sites of exons		
		Exon I	Exon II	Exon III
rps12	(-)	70853 – 70966	98776 – 99033	
rpoC1	(+)	39715 – 40144	40839 – 42463	
atpF	(+)	50529 – 50695	51336 – 51747	
petB	(+)	75086 – 75091	75891 – 76532	
petD	(+)	76774 – 76781	77489 – 77963	
rpl16	(-)	83463 – 83471	81968 – 82366	
rpl2	(-)	86171 – 86569	85034 – 85468	
ndhB	(-)	97168 – 97890	95727 – 96482	
ndhA	(+)	117077 – 117627	118834 – 119374	
trnK-UUU	(-)	4333 – 4369	1762 – 1796	
trnV-UAC	(+)	10010 – 10047	10621 – 10657	
trnL-UAA	(-)	14611 – 14647	14065 – 14114	
trnG-UCC	(-)	54778 – 54800	54043 – 54093	
trnI-GAU	(+)	102778 – 102819	103599 – 103633	
trnA-UGC	(+)	103702 – 103739	104543 – 104579	
ycf3	(+)	17796 – 17919	18634 – 18861	19598 – 19752
